# Human Endogenous Retroviruses and Epigenetic Regulators Are Dysregulated in Beckwith–Wiedemann Syndrome

**DOI:** 10.3390/cimb48030328

**Published:** 2026-03-19

**Authors:** Ilaria Galliano, Pier-Angelo Tovo, Cristina Calvi, Anna Pau, Anna Clemente, Paola Montanari, Stefano Gambarino, Alessandro Mussa, Massimiliano Bergallo

**Affiliations:** 1 Department of Public Health and Pediatric Sciences, University of Turin, Piazza Polonia 94, 10126 Turin, Italy; pierangelo.tovo@unito.it (P.-A.T.); cristina.calvi@unito.it (C.C.); pauanna1@gmail.com (A.P.); anna.clemente@unito.it (A.C.); paola.montanari@unito.it (P.M.); stefano.gambarino@unito.it (S.G.); alessandro.mussa@unito.it (A.M.); 2 Pediatric Laboratory, Department of Children’s Pathology and Care, Regina Margherita Children’s Hospital, Piazza Polonia 94, 10126 Turin, Italy; 3 Pediatric Clinical Genetics, Regina Margherita Children Hospital, 10126 Turin, Italy

**Keywords:** Beckwith–Wiedemann syndrome, human endogenous retroviruses, HERV-H, HERV-K, Syncytin, TRIM28, SETDB1, epigenetics, imprinting disorders

## Abstract

Beckwith–Wiedemann syndrome (BWS) is an overgrowth disorder caused by genetic and epigenetic alterations at chromosome 11p15.5. Increasing evidence suggests that imprinting defects may be accompanied by broader epigenomic perturbations affecting repetitive elements such as human endogenous retroviruses (HERVs). We quantified the transcriptional levels of the HERV-H, HERV-K, and HERV-W-*pol* genes, the HERV-derived *env* genes, Syncytin-1 (SYN1) and Syncytin-2 (SYN2), and the epigenetic regulators, TRIM28 and SETDB1, in whole blood from children and adolescents with BWS, stratified by molecular subtype (ICR2 loss of methylation, *n* = 14; UPD(11)pat, *n* = 10), and compared with age-matched healthy controls using quantitative real-time PCR. The BWS samples showed significantly increased transcription of HERV-H and HERV-K relative to controls, whereas HERV-W was unchanged. The SYN1 transcripts were significantly higher in UPD(11)pat compared with controls, while SYN2 did not differ between groups. TRIM28 and SETDB1 were significantly overexpressed in BWS, irrespective of molecular subtype, and no significant differences were observed between ICR2 and UPD(11)pat for HERV-H, HERV-K, HERV-W, TRIM28, or SETDB1. These findings indicate selective dysregulation of endogenous retroelements and key repressors in BWS, consistent with epigenetic alterations extending beyond canonical imprinted loci.

## 1. Introduction

Beckwith–Wiedemann syndrome (BWS) is a rare congenital overgrowth disorder characterized by prenatal and postnatal macrosomia, macroglossia, visceromegaly, abdominal wall defects, lateralized overgrowth, and an increased risk of embryonal tumors, particularly Wilms tumor and hepatoblastoma [1,2,3]. The estimated incidence ranges from 1:10,000 to 1:13,700 live births, although this figure is likely underestimated due to mosaicism and clinical heterogeneity [2].

From a molecular standpoint, BWS represents a prototypical imprinting disorder caused by genetic and, more frequently, epigenetic alterations at chromosome 11p15.5, a region containing two imprinting control regions (ICR1 and ICR2) that regulate parent-of-origin-specific expression of key growth-related genes [3,4]. The most common molecular subtypes include loss of methylation at the maternal ICR2, gain of methylation at ICR1, paternal uniparental disomy of chromosome 11p15 (UPD(11)pat), and pathogenic variants in CDKN1C [3,5]. These molecular lesions show clear (epi)genotype–phenotype correlations and are associated with different cancer risks, forming the basis for risk-stratified surveillance protocols [3,5,6].

While BWS has traditionally been studied through the lens of dysregulated imprinted genes, increasing evidence suggests that molecular alterations affecting the imprinted region may reflect a more global epigenomic instability affecting additional genomic compartments, including repetitive elements [7]. Human endogenous retroviruses (HERVs) are remnants of ancient retroviral infections that account for approximately 8% of the human genome [8]. Based on sequence similarity, HERVs are classified into several families, among which HERV-H, HERV-K, and HERV-W are the most biologically relevant and transcriptionally active [9,10,11].

HERV expression is normally suppressed by epigenetic mechanisms such as DNA methylation and repressive histone marks. However, HERVs can be reactivated during early embryogenesis, cellular reprogramming, cancer, and immune-mediated diseases, indicating their sensitivity to epigenetic perturbations [12,13,14,15]. Notably, HERV-H plays a role in pluripotency networks in human embryonic stem cells, while HERV-K is among the most intact and potentially active families in the human genome [16,17,18].

Epigenetic repression of HERVs is mediated primarily by the KRAB–ZFP/TRIM28/SETDB1 axis. TRIM28 (KAP1) functions as a scaffold protein recruited by KRAB zinc-finger proteins to specific retroelement loci, where it engages SETDB1, a histone H3 lysine 9 (H3K9) methyltransferase responsible for heterochromatin formation and stable silencing [19,20,21,22]. The disruption of this pathway leads to selective derepression of specific HERV families, particularly HERV-H and HERV-K [23,24,25].

Some HERV elements have been co-opted by the host genome to perform physiological functions. Among these, Syncytin-1 (SYN1), derived from HERV-W, and Syncytin-2 (SYN2), derived from HERV-FRD, play essential roles in placental trophoblast fusion and immune tolerance [26,27,28]. Beyond placentation, altered Syncytin expression has been reported in cancer, inflammatory diseases, and neurological disorders, suggesting context-dependent regulation [29,30,31].

Importantly, several studies by Bergallo, Tovo, and colleagues have demonstrated that HERV transcription is particularly high during early life, in cord blood, and in placental tissues, and it may be modulated in immune and inflammatory conditions [32,33,34,35,36]. These observations further support the sensitivity of HERV expression to developmental and epigenetic contexts.

Given that BWS is characterized by genetic and epigenetic alterations involving the imprinted region at chromosome 11p15.5, we hypothesized that these molecular alterations may be associated with altered regulation of HERVs, HERV-derived genes, and their epigenetic repressors. To test this hypothesis, we analyzed the transcriptional levels of HERV-H-, HERV-K-, and HERV-W-*pol* genes, SYN1- and SYN2-*env* genes, and the epigenetic regulators TRIM28 and SETDB1 in whole blood samples from the BWS patients and age-matched healthy controls.

## 2. Material and Methods

### 2.1. Study Populations

Children and adolescents with a clinical and molecular diagnosis of BWS were enrolled in the study. In particular, Group A1 included patients with a hypomethylation at the maternal ICR2, and Group A2 included those with UPD(11)pat. The low number of patients affected by other genetic forms of BWS did not allow us to perform an adequate statistical analysis of their findings.

The asymptomatic subjects of comparable age who were tested at the Regina Margherita Children’s Hospital, Turin, Italy, during routine laboratory examinations, whose results were all within the normal reference range, served as the control group (Group B). The subjects with any confirmed or suspected disease, such as infections, cancer, autoimmune disorders, inflammatory diseases, neurological disturbances, or abnormal laboratory results, were excluded from the study. The tests were performed using leftovers of the laboratory samples after informed parent’s consent; the data were gathered anonymously.

The clinical data were treated in accordance with the principles of the Helsinki Declaration (World Medical Association General Assembly, Seoul, Republic of Korea, October 2008). The study protocol was approved by the ethics committee of Azienda Ospedaliera-Universitaria Città della Salute e della Scienza, Turin (code 1103 del 8 August 2019).

### 2.2. Molecular Assay for Methylation Anomalies of BWS

The molecular assay for testing methylation anomalies of BWS was performed through methylation-sensitive multiple ligation probe amplification (SALSA^®^ MS-MLPA^®^ Probemix ME030-C3 BWS/SRS) for the 11p15.5 region (MRC Holland, Willem Schoutenstraat Amsterdam, The Netherlands). UPD(11)pat was confirmed through the single nucleotide polymorphisms (SNP array) analysis.

### 2.3. Total RNA Extraction and Retro-Transcription

The total RNA was extracted from whole blood using the automated extractor, Maxwell (Promega, Madison, WI, USA), following the RNA Blood Kit protocol without modification. This kit provides treatment with DNase during the RNA extraction process. Four hundred nanograms of the total RNA was reverse-transcribed with 2 μL of buffer 10X, 4.8 μL of MgCl_2_ 25 mM, 2 μL ImpromII (Promega), 1 μL of RNase inhibitor 20 U/L, 0.4 μL random hexamers 250 μM (Promega), 2 μL mix dNTPs 100 mM (Promega), and dd-water in a final volume of 20 μL. The reaction mix was carried out in a GeneAmp PCR system 9700 thermal cycler (Applied Biosystems, Foster City, CA, USA) under the following conditions: 5 min at 25 °C, 60 min at 42 °C, and 15 min at 70 °C for the inactivation of enzyme; the cDNAs were stored at −20° until use.

### 2.4. Transcription Levels of HERV-H, -K, and -W pol Genes, SYN1 and SYN2 of env Genes, and of TRIM28 and SETDB1

Relative quantification (RE) of the transcription levels of *pol* genes of HERV-H, HERV-K, HERV-W, of *env* genes of SYN1, SYN2, and of TRIM28 and SETDB1 were achieved using the primers and probes reported in Table 1.

Briefly, 40 ng of cDNA were amplified in a 20 μL total volume reaction, containing 2.5 U goTaQ MaterMix (Promega), 1.25 mmol/L MgCl2, 500 nmol of specific primers, and 200 nmol of specific probes. All amplifications were run in a 96-well plate at 95 °C for 10 min, followed by 45 cycles at 95 °C for 15 s and at 60 °C for 1 min. Each sample was run in triplicate. Relative quantification of target gene transcripts was performed according to the 2^−ΔΔCt^ method. Briefly, after normalization of the PCR result of each target gene with the housekeeping gene (GAPDH), the method includes additional calibration using the expression of the target gene evaluated in a pool of healthy controls. The results, expressed in arbitrary units (called relative quantification, RE), show the variations in target gene transcripts relative to the standard set of controls. Since we measured Ct for every target in all samples, we argue that our methods were suitable for HERV, TRIM28, and SETDB1 detection and quantification. For control of genomic DNA contamination, we directly amplified the RNA extracts without reverse transcription.

All analyses were performed in a laboratory of biosafety level 2 (BSL-2), according to the National Institutes of Health (NIH) and WHO guidelines [37,38].

### 2.5. Statistical Analysis

Kruskal–Wallis test, followed by Dunn’s multiple comparisons test, was used to compare the transcriptional levels of each target between the groups of children. The statistical analyses were done using the Prism software 9.0 (GraphPad Software, La Jolla, CA, USA). In all analyses, *p* < 0.05 was taken to be statistically significant.

## 3. Results

### 3.1. Study Population

The characteristics of BWS patients and healthy controls (HCs) are reported in Table 2. HCs were subdivided into two subgroups according to the tests performed: Group B1 were evaluated for *pol* genes of HERV-H, -K, and -W; Group B2 were evaluated for *env* genes of Syn1 and SYn2, as well as for TRIM28 and SETDB1. The age comparison across the four groups was performed using the Kruskal–Wallis test and showed no statistically significant differences (*p* = 0.2955), confirming that the groups were age-matched.

### 3.2. Expression Levels of Housekeeping Gene

The expression of the housekeeping gene, GAPDH, was similar between BWS patients and age-matched HCs. The median and IQR 25–75% values were: Group A1 21.8, 21.2–22.1; Group A2 21.9, 21.6–22.1; Group B1 21.7, 21.0–22.3; and Group B2 21.8, 21.4–22.3 (A1 vs. A2 *p* = 0.9771; A1 vs. B1 *p* = 0.5886; A1 vs. B2 *p* = 0.2878; A2 vs. B1 *p* = 0.6038; A2 vs. B2 *p* = 0.3086; and B1 vs. B2 *p* = 0.3071).

### 3.3. Expressions of HERV-H-pol, HERV-K-pol, and HERV-W-pol

**Figure 1:**
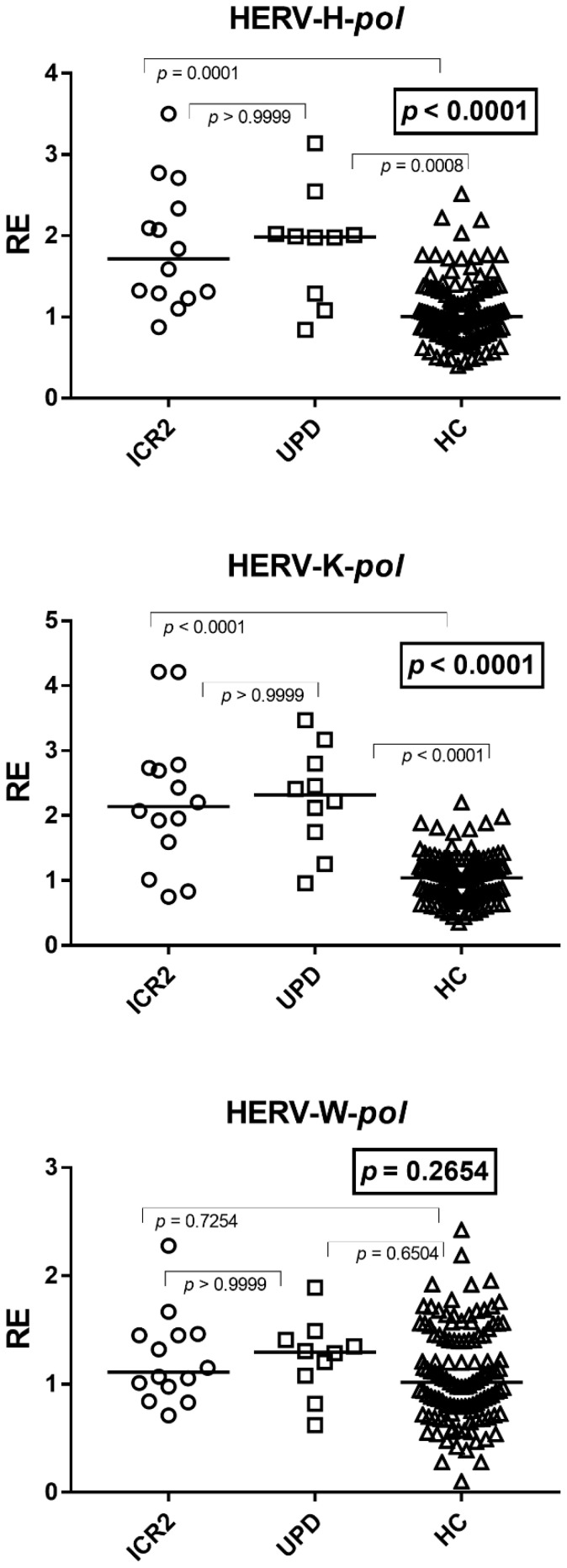
Transcription levels of *pol* genes of HERV-H, HERV-K, and HERV-W in whole blood from 14 BWS patients with hypomethylation at the maternal ICR2 (ICR2), 10 BWS patients with UPD(11)pat (UPD), and 118 age-matched healthy controls (HCs). RE: relative expression, normalized to GAPDH and calibrated against the healthy control pool according to the 2^−ΔΔCt^ method. Circles, squares, and triangles show the median of three individual measurements, and horizontal lines show the median values. Statistical analysis: Kruskal–Wallis test (boxed *p* values) followed by Dunn’s multiple comparisons test.

The transcription levels of *pol* genes of HERV-H and HERV-K were significantly higher in subjects with BWS as compared to age-matched HCs, while no difference was found for HERV-W (Figure 1). In particular, the median and IQR of HERV-H-*pol* values were: Group A1 (ICR2 patients) 1.71, 1.30–2.27; Group A2 (UPD(11)pat) patients 1.99, 1.46–2.02; Group B1 1.01, 0.79–1.33; those of HERV-K-*pol* values were: Group A1 2.14, 1.68–2.73; Group A2 2.32, 1.84–2.; and Group B1 1.05, 0.79–1.28; and those of HERV-W-*pol* values were: Group A1 1.11, 0.98–1.45; Group A2 1.30, 1.11–1.39; and Group B1 1.02, 0.79–1.45. No difference in HERV-*pol* genes were observed between Group A1 and Group A2 patients (Figure 1).

### 3.4. Expressions of SYN1-env and SYN2-env

**Figure 2:**
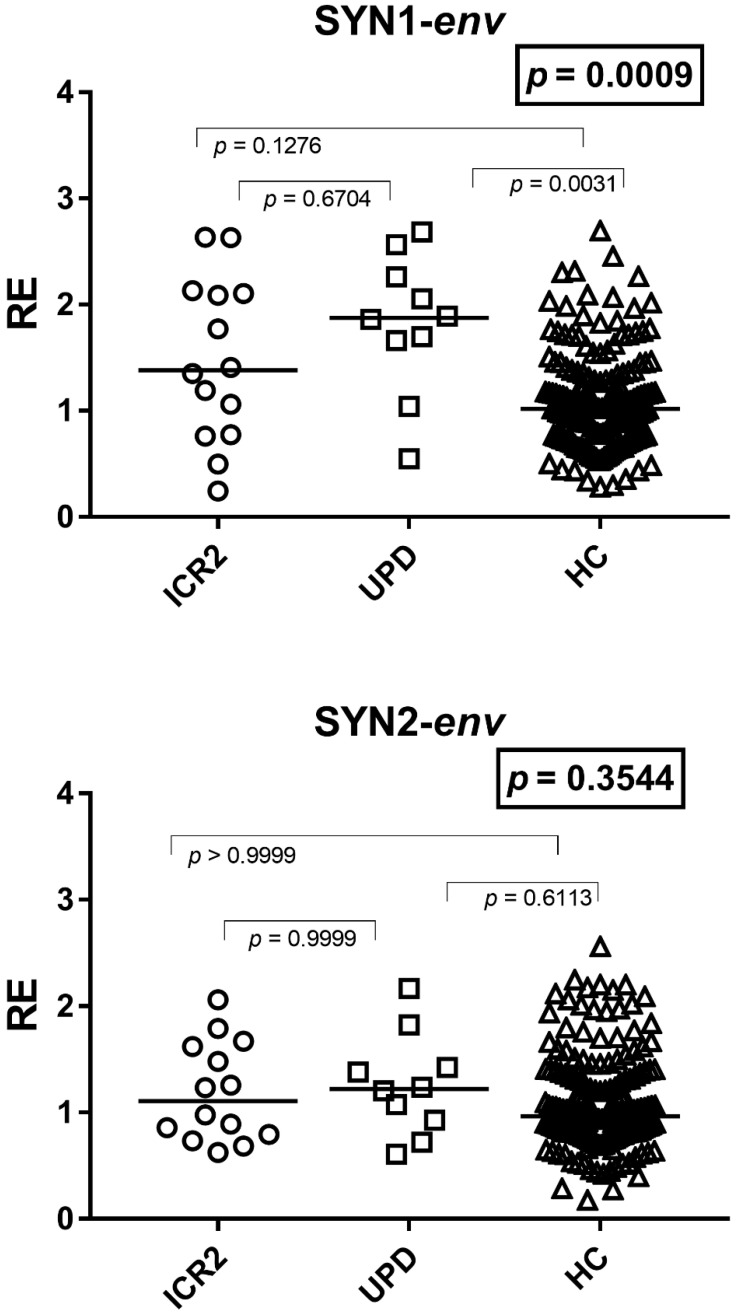
Transcription levels of *env* genes of Syncytin-1 and Syncytin-2 in whole blood from 14 BWS patients with hypomethylation at the maternal ICR2 (ICR2), 10 BWS patients with UPD(11)pat (UPD), and 90 age-matched healthy controls (HCs). RE: relative expression, normalized to GAPDH and calibrated against the healthy control pool according to the 2^−ΔΔCt^ method. Circles, squares, and triangles show the median of three individual measurements, and horizontal lines show the median values. Statistical analysis: Kruskal–Wallis test (boxed *p* values) followed by Dunn’s multiple comparisons test.

The transcription levels of *env* genes of SYN1 were significantly higher in subjects with UPD(11)pat as compared to age-matched HCs, while no difference was found between the ICR2 subjects and HCs and between the two groups of BWS patients. No differences were found for SYN2 (Figure 2). In particular, the median and IQR of SYN1 were: Group A1 (ICR2) 1.38, 0.85–2.10; Group A2 (UPD(11)pat) 1.88, 1.67–2.21; and Group B2 1.02, 0.74–1.36; the median and IQR of SYN2-*env* values were: Group A1 1.11, 0.81–1.59; Group A2 1.22, 0.96–1.41; and Group B2 0.96, 0.81–1.34.

### 3.5. Expressions of TRIM28 and SETDB1

**Figure 3:**
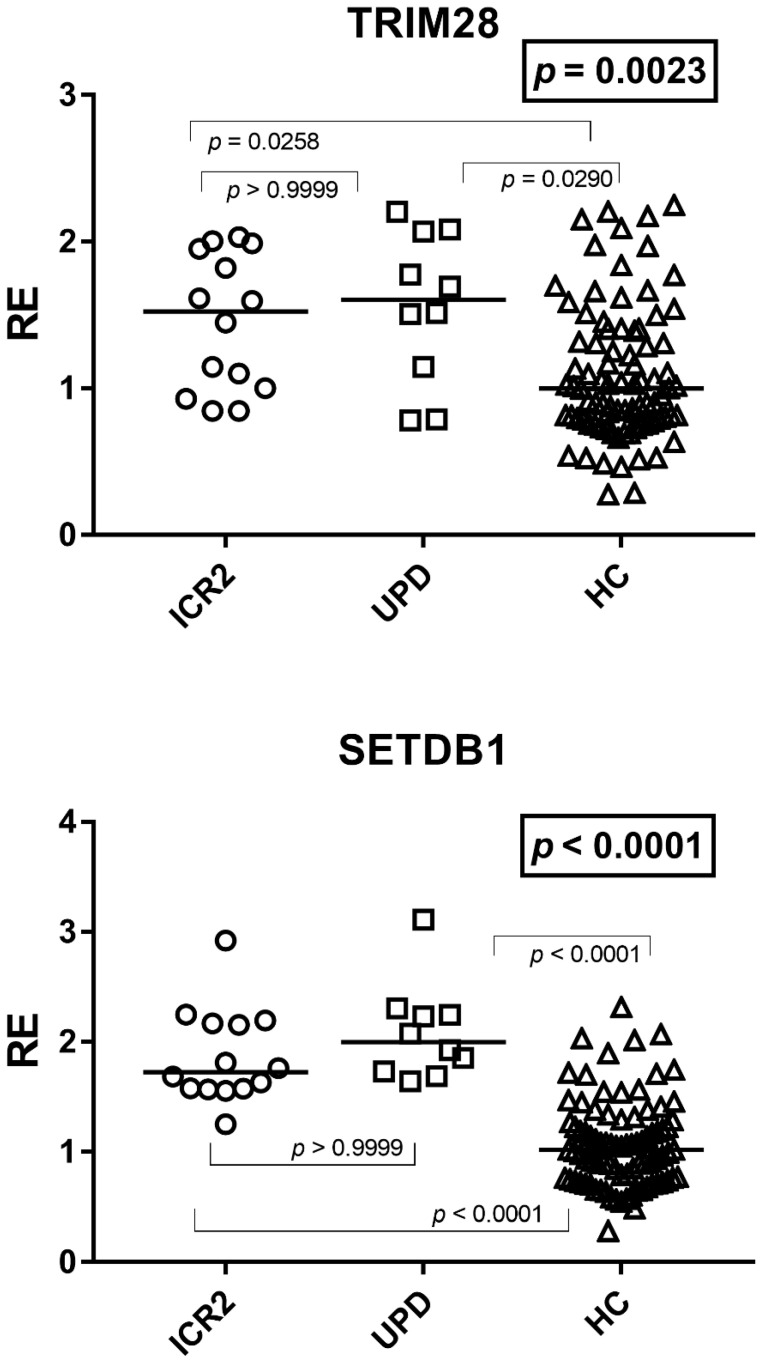
Transcription levels of TRIM28 and SETDB1 in whole blood from 14 BWS patients with hypomethylation at the maternal ICR2 (ICR2), 10 BWS patients with UPD(11)pat (UPD), and 90 age-matched healthy controls (HCs). RE: relative expression, normalized to GAPDH and calibrated against the healthy control pool according to the 2^−ΔΔCt^ method. Circles, squares, and triangles show the median of three individual measurements, and horizontal lines show the median values. Statistical analysis: Kruskal–Wallis test (boxed *p* values) followed by Dunn’s multiple comparisons test.

The expressions of TRIM28 and SETDB1 were significantly higher in both groups of BWS patients as compared to HCs, while no difference emerged between Group A1 and Group A2 patients (Figure 3). In particular, the median and IQR of TRIM28 values were: Group A1 (ICR2 patients) 1.52, 1.03–1.92; Group A2 (UPD(11)pat patients 1.60, 1.24–2.00; and Group B2 (HC) 1.00, 0.79–1.37. The median and IQR of SETDB1 values were: Group A1 1.72, 1.58–2.17; Group A2 2.00, 1.76–2.24; and Group B2 1.02, 0.75–1.28.

## 4. Discussion

This study demonstrates that Beckwith–Wiedemann syndrome (BWS) is associated with a selective dysregulation of human endogenous retroviruses (HERVs) and the epigenetic machinery involved in their repression. Specifically, we observed increased transcriptional activity of HERV-H and HERV-K in patients with BWS, unchanged expression of HERV-W, subtype-specific upregulation of the HERV-derived gene Syncytin-1 in patients with paternal uniparental disomy of chromosome 11p15 (UPD(11)pat), stable levels of Syncytin-2, and a significant increase in the expression of the epigenetic repressors TRIM28 and SETDB1 in both major molecular subtypes of BWS. Collectively, these findings suggest that the molecular alterations involving the 11p15.5 imprinted region in BWS may be associated with broader epigenomic changes extending beyond canonical imprinted loci. The primers used for HERV-H-*pol* and HERV-K-*pol* amplify conserved regions present in multiple genomic copies dispersed throughout the genome rather than loci specifically located near chromosome 11p15.5. Thus, the observed transcriptional alterations are consistent with the cumulative expression of widely distributed retroelement families rather than local cis-effects associated with the imprinted region. In addition, Syncytin-1 is located on chromosome 7q21.2, further supporting the interpretation that these findings are consistent with broader epigenomic perturbations rather than locus-dependent changes.

The increased expression of HERV-H and HERV-K is particularly noteworthy, given the biological properties of these families and their known sensitivity to epigenetic perturbations. HERV-H elements are highly expressed during early human embryogenesis and actively participate in pluripotency-associated transcriptional networks in human embryonic stem cells, where they contribute to chromatin organization and gene regulation [16,17]. Their aberrant expression in peripheral blood from BWS patients may therefore reflect a persistent relaxation of developmental epigenetic programs, consistent with the congenital and growth-related nature of the disorder [1,2,3]. Similarly, HERV-K represents the most evolutionarily recent and transcriptionally competent HERV family in the human genome and is frequently reactivated in pathological contexts characterized by epigenetic instability, including cancer and developmental disorders [11,18,24]. The concomitant upregulation of both HERV-H and HERV-K suggests that these families are particularly vulnerable to the epigenetic disturbances associated with BWS.

In contrast, HERV-W-pol transcription was not significantly altered in BWS patients, underscoring the family- and locus-specific nature of endogenous retrovirus regulation [9,12,13]. This observation indicates that the epigenetic alterations underlying BWS do not result in a generalized derepression of repetitive elements but rather selectively affect subsets of HERVs depending on genomic context, regulatory architecture, and chromatin environment. Such selectivity is consistent with previous reports demonstrating that distinct HERV families respond differently to changes in DNA methylation and histone modifications [9,10,12].

Despite stable HERV-W-pol expression, we observed a significant increase in Syncytin-1 transcription, specifically in patients with UPD(11)pat, while no significant changes were detected in patients with ICR2 hypomethylation or in Syncytin-2 expression. This dissociation highlights the importance of distinguishing between global HERV family activity and the regulation of individual HERV-derived genes. Syncytin-1 is controlled by promoter-specific epigenetic mechanisms and transcription factors that differ from those regulating other HERV-W loci [26,27,30]. Its selective upregulation in UPD(11)pat patients suggests that subtype-specific imprinting defects may influence the regulation of co-opted retroviral genes independently of broader HERV family activity. By contrast, the stability of Syncytin-2 expression is consistent with its distinct evolutionary origin and regulatory landscape [28].

Although Syncytins are classically associated with placental development and trophoblast fusion [26,27,28], increasing evidence indicates that they may exert broader biological effects, including modulation of immune responses and cell–cell fusion processes beyond the placenta [29,30,31]. Altered Syncytin-1 expression in BWS, particularly in UPD(11)pat patients, may therefore contribute to systemic aspects of the disorder, potentially influencing immune homeostasis or tissue interactions. While our analyses were limited to peripheral blood, these findings raise the possibility that Syncytin dysregulation may also be present in other tissues relevant to BWS pathophysiology.

A central and unexpected finding of this study is the increased expression of TRIM28 and SETDB1 in BWS patients, despite the concomitant upregulation of HERV-H and HERV-K. TRIM28 (KAP1) and SETDB1 are core components of the KRAB zinc-finger protein-mediated repression pathway that establishes and maintains heterochromatin at endogenous retroelement loci through H3K9 trimethylation [19,20,21,22]. Increased transcription of these factors would, in principle, be expected to reinforce retroelement silencing. The observed coexistence of elevated TRIM28/SETDB1 expression and increased HERV transcription therefore suggests a functional uncoupling between the abundance of epigenetic repressors and their effective activity at specific retroelement loci.

Several non-mutually exclusive mechanisms may account for this apparent paradox. First, increased TRIM28 and SETDB1 expression may represent a compensatory response to primary epigenetic instability, aimed at restoring heterochromatin integrity. Second, imprinting defects at 11p15.5 may indirectly affect chromatin organization, KRAB–ZFP targeting, or higher-order nuclear architecture, thereby limiting the ability of TRIM28 and SETDB1 to effectively silence specific HERV families. Third, quantitative increases in epigenetic repressors may not translate into functional repression if recruitment to target loci is impaired or if local chromatin states are refractory to re-establishment of heterochromatin. Similar dissociations between repressor expression and retroelement silencing have been reported in experimental models and in immune-mediated or inflammatory conditions characterized by epigenetic dysregulation [23,24,25,32,33,34,35,36].

However, since the present study is limited to transcriptional analyses, the observed expression patterns of TRIM28 and SETDB1 cannot be directly linked to functional changes in heterochromatin organization or retroelement repression and should be interpreted as transcriptional correlates of epigenetic perturbation rather than evidence of causal mechanisms.

Importantly, increased transcription of TRIM28 and SETDB1 does not necessarily imply a proportional increase in protein abundance or repressive activity. It is well-established that transcript levels do not invariably correlate with protein levels due to extensive post-transcriptional and post-translational regulation [39]. In addition, TRIM28 activity is modulated by post-translational modifications such as phosphorylation and SUMOylation, which influence its chromatin recruitment and co-repressor function within the KRAB-ZFP/TRIM28/SETDB1 axis [40]. Therefore, the present findings should be interpreted as transcriptional indicators of epigenetic perturbation rather than direct evidence of increased functional repression at the protein level. Further studies integrating protein-based and chromatin-level analyses will be necessary to clarify the functional consequences of these alterations in BWS.

Importantly, with the exception of Syncytin-1, whose expression was increased in UPD(11)pat patients compared with healthy controls but did not differ significantly between BWS molecular subtypes, we did not observe significant differences in HERV expression or in TRIM28 and SETDB1 levels between the two major molecular subgroups. This suggests that distinct primary molecular alterations, namely ICR2 hypomethylation and UPD(11)pat, converge on shared downstream epigenetic alterations affecting repetitive element regulation. Such convergence mirrors previous observations that different molecular subgroups of BWS share overlapping clinical features and tumor risks despite heterogeneous initiating lesions [3,5,6]. At the same time, the subtype-specific regulation of Syncytin-1 indicates that certain downstream effects remain sensitive to the nature of the underlying imprinting defect. Although no significant differences were detected between the two molecular subtypes, the relatively limited sample size may have reduced the power to identify subtle subtype-specific effects. Accordingly, the apparent convergence of downstream HERV expression patterns should be interpreted cautiously.

From a clinical perspective, the derepression of endogenous retroviruses may have implications for tumor susceptibility in BWS. HERV overexpression and retroviral protein production have been implicated in oncogenesis [41,42]. Moreover, endogenous retroelements are physiologically active during early embryogenesis and in pluripotent cells [17], developmental contexts that are also relevant to the embryonal histotypes observed in BWS-associated tumors. Given that tumor predisposition in BWS is primarily associated with the UPD(11)pat molecular subtype, persistent dysregulation of HERVs in this subgroup may represent one component of a broader epigenetic instability potentially relevant to tumor susceptibility. However, this hypothesis remains speculative and requires further investigation.

Conceptually, our findings support the view that Beckwith–Wiedemann syndrome may be associated with broader alterations of epigenomic architecture rather than a condition confined to dysregulation of imprinted genes at chromosome 11p15.5. In this framework, endogenous retroelements emerge not merely as passive indicators of epigenetic disturbance but as dynamic genomic components that may amplify or modulate disease-relevant transcriptional states. This perspective aligns with emerging views of transposable elements as integral contributors to developmental gene regulation and disease susceptibility, particularly in conditions originating from early embryonic epigenetic perturbations [40,43,44].

### Limitations and Future Directions

A limitation of this study is that transcriptional analyses were performed in peripheral blood rather than in tissues directly involved in BWS-associated overgrowth or tumorigenesis. However, obtaining biopsies from affected tissues in pediatric patients, and especially from healthy control children, raises ethical and practical concerns. Peripheral blood therefore represents a minimally invasive and ethically acceptable surrogate tissue for investigating systemic epigenetic alterations [1,2,5]. While blood-based analyses allow the detection of constitutional or systemic transcriptional signatures, they may not fully recapitulate tissue-specific epigenetic changes occurring in overgrown organs or tumor-prone tissues. The detection of HERV dysregulation in peripheral hematopoietic cells suggests that epigenetic alterations in BWS may extend beyond tissue-specific overgrowth and involve systemic transcriptional signatures. However, it remains unclear whether these changes reflect a primary constitutional epigenetic defect established during early development or whether they are secondary to systemic growth factor imbalances characteristic of BWS, such as altered IGF2 signaling. Future studies integrating tissue-specific analyses, genome-wide epigenomic profiling, and longitudinal follow-up will be essential to clarify the biological and clinical relevance of HERV dysregulation and to determine whether endogenous retroelements may serve as biomarkers or modifiers of disease risk within the Beckwith–Wiedemann spectrum.

## 5. Conclusions

In conclusion, this study shows that Beckwith–Wiedemann syndrome (BWS) is associated with selective alterations in the transcriptional activity of endogenous retroelements and of key epigenetic regulators involved in their repression. Patients with BWS display increased expression of HERV-H and HERV-K, unchanged HERV-W transcription, increased Syncytin-1 expression in patients with paternal uniparental disomy of chromosome 11p15 (UPD(11)pat) compared with healthy controls, stable Syncytin-2 levels, and increased transcription of the epigenetic repressors, TRIM28 and SETDB1.

These findings indicate that molecular alterations involving the 11p15.5 imprinted region in BWS are accompanied by broader transcriptional changes affecting repetitive elements, extending beyond canonical imprinted loci at chromosome 11p15.5 [3,5,7]. The selective nature of HERV dysregulation, together with the differential behavior of HERV-derived genes, highlights the locus- and family-specific response of endogenous retroviruses to epigenetic perturbations [9,12,16,26,27,28].

Importantly, the concomitant increase in TRIM28 and SETDB1 expression observed alongside elevated HERV-H and HERV-K transcription suggests that transcriptional upregulation of epigenetic repressors does not necessarily translate into effective silencing of specific retroelement families. While these results are consistent with an altered epigenetic landscape in BWS, the present study is limited to transcriptional analyses and does not allow direct inference of functional interactions or causal mechanisms linking molecular alterations affecting the 11p15.5 imprinted region, epigenetic regulators, and retroelement activity [19,20,21,22,23,24,25].

Overall, our results support the view that Beckwith–Wiedemann syndrome is associated with widespread transcriptional signatures of epigenetic perturbation rather than being restricted to the dysregulation of imprinted genes alone. In this context, endogenous retroelements may represent sensitive transcriptional readouts of epigenomic instability in imprinting disorders, warranting further investigation through functional and genome-wide epigenomic approaches [40,43,44].

## Figures and Tables

**Table 1:** Primers and probes used to assess the transcription levels of *pol* genes of HERV-H, -K, and -W, of *env* genes of Syncytin-1, Syncytin-2, of TRIM28 and SETDB1, and of GAPDH.

Name	Primer/ Probe	Sequence
HERV-H-*pol*	Forward	5′-TGGACTGTGCTGCCGCAA-3′
	Reverse	5′-GAAGSTCATCAATATATTGAATAAGGTGAGA-3′
	Probe	6FAM-5′-TTCAGGGACAGCCCTCGTTACTTCAGCCAAGCTC-3′-TAMRA
HERV-K-*pol*	Forward	5′-CCACTGTAGAGCCTCCTAAACCC-3′
	Reverse	5′-TTGGTAGCGGCCACTGATTT-3′
	Probe	6FAM-5′-CCCACACCGGTTTTTCTGTTTTCCAAGTTAA-3′-TAMRA
HERV-W-*pol*	Forward	5′-ACMTGGAYKRTYTTRCCCCAA-3′
	Reverse	5′-GTAAATCATCCACMTAYYGAAGGAYMA-3′
	Probe	6FAM-5′-TYAGGGATAGCCCYCATCTRTTTGGYCAGGCA-3′-TAMRA
Syncytin-1 *env*	Forward	5′-ACTTTGTCTCTTCCAGAATCG-3′
	Reverse	5′-GCGGTAGATCTTAGTCTTGG-3′
	Probe	6FAM-5′-TGCATCTTGGGCTCCAT-3′-TAMRA
Syncytin-2 *env*	Forward	5′-GCCTGCAAATAGTCTTCTTT-3′
	Reverse	5′-ATAGGGGCTATTCCCATTAG-3′
	Probe	6FAM-5′-TGATATCCGCCAGAAACCTCCC-3′-TAMRA
TRIM28	Forward	5′-GCCTCTGTGTGAGACCTGTGTAGA-3′
	Reverse	5′-CCAGTAGAGCGCACAGTATGGT-3′
	Probe	6FAM-5′-CGCACCAGCGGGTGAAGTACACC-3′-TAMRA
SETDB1	Forward	5′-GCCGTGACTTCATAGAGGAGTATGT-3′
	Reverse	5′-GCTGGCCACTCTTGAGCAGTA-3′
	Probe	6FAM-5′-TGCCTACCCCAACCGCCCCAT-3′-TAMRA
GAPDH	Forward	5′-CGAGATCCCTCCAAAATCAA-3′
	Reverse	5′-TTCACACCCATGACGAACAT-3′
	Probe	6FAM-5′-TCCAACGCAAAGCAATACATGAAC-3′-TAMRA

**Table 2:** Demographic and clinical characteristics of subgroups of patients affected by Beckwith–Wiedemann (BWS) and healthy children. IQR: interquartile range, expressed as 25 and 75 quartile values.

BWS Patients		Healthy Children (HC)	
Group A1	Group A2	Group B1	Group B2
Hypomethylation at the maternal ICR2	UPD(11)pat	Tested for *pol* genes of HERV-H, -K, and -W	Tested for *env* genes of Syn1 and Syn2, for TRIM28 and SETDB1
n. 14	n. 10	n. 118	n. 90
Males	Males	Males	Males
*n* (%): 4 (28.6)	*n* (%): 7 (70.0)	*n* (%): 62 (52.5)	*n* (%): 55 (61.1)
Age	Age	Age	Age
Median (IQR):	Median (IQR):	Median (IQR):	Median (IQR):
8.4 (4.0–15.4) years	12.3 (6.4–15.7) years	5.5 (3.8–11.9) years	6.3 (4.0–11.3) years

## Data Availability

The data presented in this study are available on request from the corresponding authors (the data are not publicly available due to privacy restrictions).
